# Endogenous Secretory Receptor for Advanced Glycation End Products Protects Endothelial Cells from AGEs Induced Apoptosis

**DOI:** 10.1155/2018/8216578

**Published:** 2018-04-10

**Authors:** Guomin Yang, Yinqiong Huang, Xiaohong Wu, Xiahong Lin, Jinting Xu, Xiaoyu Chen, Xuefeng Bai, Qiulan Li

**Affiliations:** ^1^Department of Endocrinology, Second Affiliated Hospital of Fujian Medical University, Quanzhou, Fujian 362000, China; ^2^Scientific Research Center, Second Affiliated Hospital of Fujian Medical University, Quanzhou, Fujian 362000, China

## Abstract

Endogenous secretory receptor for advanced glycation end products (esRAGE) binds extracellular RAGE ligands and blocks RAGE activation on the cell surface, protecting endothelial cell function. However, the underlying mechanism remains unclear. Endothelial cells overexpressing the esRAGE gene were generated using a lentiviral vector. Then, quantitative real-time polymerase chain reaction (qRT-PCR) and enzyme-linked immunosorbent assay (ELISA) were used to assess esRAGE mRNA and protein levels, respectively. Hoechst-PI double staining was used to assess apoptosis. Western blot and qRT-PCR were used to assess the expression levels of apoptosis-related factors and the proinflammatory cytokine NF-кB. Compared with the control group, AGEs significantly induced endothelial cell apoptosis, which was significantly reduced by esRAGE overexpression. Incubation with AGEs upregulated the proapoptotic factor Bax and downregulated the antiapoptotic factor Bcl-2. Overexpression of esRAGE reduced Bax expression induced by AGEs and increased Bcl-2 levels. Furthermore, AGEs increased the expression levels of proinflammatory cytokine NF-кB, which were reduced after esRAGE overexpression. esRAGE protects endothelial cells from AGEs associated apoptosis, by downregulating proapoptotic (Bax) and inflammatory (NF-кB) factors and upregulating the antiapoptotic factor Bcl-2.

## 1. Introduction

Chronic complications of diabetes severely affect the quality of life and causes death, imposing serious socioeconomical burden on individuals, families, and society [1]. Major blood vessel disease and microangiopathy constitute the common pathological basis of chronic complications of diabetes, and are initiated by endothelial cell injury [2]. Among the multiple physiopathological etiologies of chronic complications of diabetes, formation of nonenzymatic glycation and advanced glycation end products (AGEs) represents one of the main mechanisms [3]. AGEs cause endothelial cell injury by binding RAGE on the cell surface, which leads to the activation of a series of intracellular signaling molecules; this in turn results in NF-*κ*B upregulation and increased inflammatory response [4]. Meanwhile, oxidative stress is increased, as well as reactive oxygen species (ROS) levels, with reduced superoxide dismutase (SOD) activity, which promotes apoptosis by increasing the expression levels of proapoptotic proteins (e.g., cytochrome C, caspase-9, caspase-3, and Bax) and decreasing Bcl-2 (an antiapoptotic protein) expression [5–11].

RAGE is widely distributed in various human cells and significantly upregulated under pathological conditions, such as diabetes, cardiovascular diseases, and inflammation, promoting rapid development of arteriosclerosis [12, 13]. RAGE has 3 splice variants, including full-length membrane bound, N-truncated membrane bound, and C-truncated soluble types [14]. In humans, soluble RAGE mainly encompasses two components, including esRAGE (spliceosome variant) and sRAGE, which is generated by metalloprotease degradation [14–16]. esRAGE accounts for 20–50% of the soluble RAGE [17]. Firstly found in endothelial cells, esRAGE is considered a decoy receptor, captures extracellular RAGE ligands, blocks RAGE activation on the cell surface [14, 18], neutralizes VEGF upregulation by AGEs, and inhibits AGEs associated endothelial cell proliferation as well as the formation of chain-like structure of endothelial cells, protecting endothelial cell function and reducing AGEs associated vascular injury [14]. However, clinical trials have yielded controversial findings. On one hand, studies found that low plasma esRAGE levels are associated with diabetic arteriosclerosis [19–21] as well as metabolic syndrome and arteriosclerosis in the prediabetes group [17, 22]. Moreover, others reported that esRAGE levels are not correlated with diabetic arteriosclerosis, although high esRAGE content is a predictive factor of cardiovascular diseases [23–25]. The protective effects of esRAGE mainly include antioxidative stress, anti-inflammation, antiplatelet activation, and impact on other arteriosclerosis-related factors such as obesity and blood fat [21, 22, 26–28]. However, the protective effects of esRAGE on endothelial cells have not been fully understood.

Therefore, the present study aimed to assess the effects of esRAGE on AGEs associated apoptosis in endothelial cells. In the human umbilical vein endothelial cells (HUVECs) model, esRAGE overexpression conferred protective effects from AGEs associated apoptosis, by downregulating Bax and NF-кB and upregulating Bcl-2.

## 2. Material and Methods

### 2.1. Cell Culture

HUVEC were obtained from the American Type Culture Collection (ATCC) and cultured in RPMI 1640 (Gibco, USA) supplemented with 10% FBS (Gibco, USA) and 1% penicillin-streptomycin (Hyclone, USA). HEK-293T cells were obtained from the Chinese Academy of Sciences (Shanghai, China) and cultured in DMEM (Gibco, USA) containing 10% FBS and 1% penicillin-streptomycin. All cells were maintained in humidified atmosphere containing 5%  CO_2_ at 37°C.

Stably transfected HUVEC overexpressing esRAGE were generated using the overexpression plasmid vector pGV367 (Addgene); esRAGE was amplified using the following primers (Shanghai Gene Company, Shanghai, China): forward, 5′-TGCCTAATGAGAAGGGAGTATC-3′; reverse, 5′-AGCTACAGGAGAAGGTGGGAC-3′. The amplified sequences were inserted into pGV367 by the recombinant method (Hanbio, Shanghai, China) according to the manufacturer's instructions. The resultant plasmid was then transfected into HEK-293T cells to construct a lentivirus overexpressing esRAGE.

HUVEC at 1 × 10^5^/well (6 well plates) were seeded and cultured for 12 h. A volume of 2 mL enhanced infection solution containing Lv-esRAGE (Shanghai Gene Company, Shanghai, China) with the corresponding viral load and control virus expressing green fluorescent protein (GFP) with puromycin acetyltransferase was incubated with cells. At 12 h after infection, the medium was replaced by the conventional culture medium. After 72 h, GFP expression was observed under a fluorescence microscope (ZEISS, Germany). Complete medium containing 2 *μ*g/mL puromycin (Shanghai Sangon Biological Engineering Technology & Services Co., Ltd., China) was used to screen virus infected cells. Afterward, 1 *μ*g/mL was used for further screening. Quantitative real-time polymerase chain reaction (qRT-PCR) was used to assess relative expression levels of esRAGE mRNA in the Lv-esRAGE infection group.

### 2.2. Cell Apoptosis

Complete culture media containing 50 *μ*g/ml, 100 *μ*g/ml, and 200 *μ*g/ml AGE-BSA (Biovision, USA), respectively, were used to culture HUVEC for 24 h [7–10]. Complete medium containing 200 *μ*g/mL AGE-BSA was used to culture Lv-esRAGE and control virus transfected cells, as well as nontransfected cells for 24 h. Another control group was set up with nontransfected cells cultured in complete medium. After culture, the culture medium from 96-well plates was collected and mixed with 50 *μ*L complete medium containing Hoechst (Sigma, USA; 5 mg/ml) and PI (Sigma, USA; 5 mg/ml). After incubation at 37°C for 10 min, the cells were observed under an inverted fluorescence microscope. Image-Pro-Plus 6.0 (Media Cybernetics, Rockville, Maryland, USA) was used to count Hoechst (total cells) and PI positive (apoptotic cells) spots. The apoptotic rate was then derived as apoptotic cell number/total cell number. The experiments were performed in triplicate.

### 2.3. Quantitative RT-PCR (qRT-PCR)

Total RNA from HUVECs was extracted with TRIzol method (Invitrogen, USA). RNA was reverse transcribed into cDNA using the two-step method with PrimeScript™ RT reagent kit with gDNA Eraser (Takara, China), according to the manufacturer's instructions. Then, qRT-PCR was performed with the SYBR® Premix Ex TaqTM kit (Takara, China), according to the manufacturer's protocol. The procedure was 95°C for 1 min; 95°C for 15 s and 60°C for 34 s, for 40 cycles; 95°C for 15 s, 60°C for 1 min, and 95°C for 15 s. The following primers were used: Bax, forward 5′-CCCGAGAGGTCTTTTTCCGAG-3′ and reverse 5′-CCAGCCCATGATGGTTCTGAT-3′; NF-кB, forward 5′-GTGGGGACTACGACCTGAATG-3′ and reverse 5′-GGGGCACGATTGTCAAAGATG-3′; Bcl-2, forward 5′-GGTGGGGTCATGTGTGTGG-3′ and reverse 5′-CGGTTCAGGTACTCAGTCATCC-3′; GAPDH, forward 5′-ACCCACTCCTCCACCTTTG-3′ and reverse 5′-CTCTTGTGCTCTTGCTGGG-3′.

GAPDH was used as reference gene, with the 2^−ΔΔCt^ method used for quantitation [29]. Triplicate experiments were performed and repeated at least 3 times.

### 2.4. Western Blot

RIPA lysis buffer (Beyotime, China) supplemented with 1 mM PMSF (Sigma Aldrich, UAS) was used for cell lysis. The Bradford method was used to assess protein concentration. Equal amounts of protein were separated by 10% SDS-PAGE and transferred onto PVDF membranes (Millipore, USA). After blocking with 5% skim milk at room temperature for 2 h, the membranes were incubated with primary antibodies targeting *β*-actin (mouse monoclonal antibody; CST, USA; 1 : 8000), NF-кBp65 (rabbit monoclonal antibody; CST, USA; 1 : 1000), Bcl-2 (rabbit monoclonal antibody; CST, USA; 1 : 1000), and Bax (rabbit monoclonal antibody; CST, USA; 1 : 1000) overnight at 4°C. Then, secondary antibodies (anti-rabbit or anti-mouse IgG/HRP; CST, USA; 1 : 5000) were incubated for 2 h with shaking. After enhanced chemiluminescence (ECL) (Merck Millipore, Germany) reaction, the protein bands were revealed on a Gel Imaging System (Syngene, USA); gray values were analyzed with the Image Lab software (Bio-Rad, USA), using *β*-actin as a loading control.

### 2.5. Enzyme-Linked Immunosorbent Assay (ELISA)

The levels of esRAGE in cell culture supernatants were evaluated with an ELISA kit (B-Bridge, USA), according to the manufacturer's instructions.

### 2.6. Statistical Analysis

The SPSS 22.0 software (SPSS, USA) was used for data analysis, and data were expressed as mean ± standard deviation (SD). Group pairs were compared by *t*-test, and one way analysis of variance (ANOVA) was used to compare more than two groups. *P* < 0.05 was considered statistically significant.

## 3. Results

### 3.1. Successful esRAGE Overexpression in HUVEC

**Figure 1:**
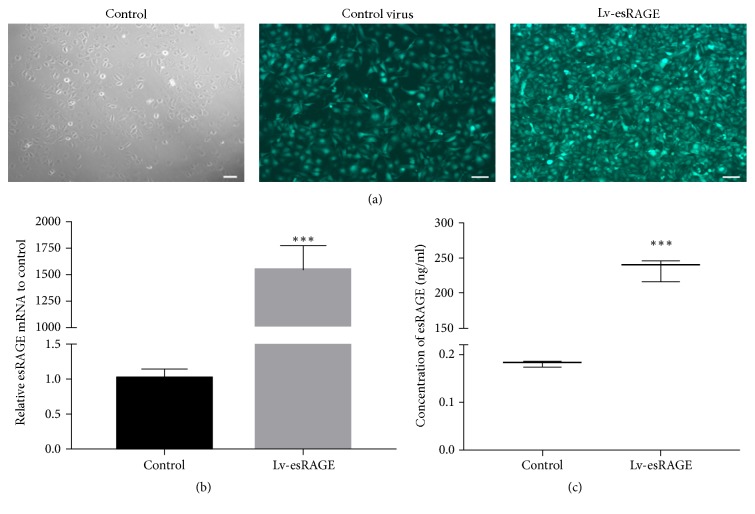
GFP expression and esRAGE expression levels after lentiviral infection in HUVEC. (a) GFP expression after HUVEC transfection with the high-expression esRAGE lentivirus and control virus. (b) esRAGE mRNA expression levels, before and after HUVEC transfection with high-expression esRAGE lentivirus. (c) esRAGE protein levels in cell culture supernatants, after HUVEC transfection with high-expression esRAGE lentivirus, ^*∗∗∗*^*P* < 0.001.

At 72 h after transfection with high-expression esRAGE lentivirus and control virus, HUVEC were observed by fluorescence microscopy. Green fluorescent protein was expressed by more than 80% of the cells (Figure 1(a)). After stable transfection and puromycin screening, esRAGE mRNA levels in the Lv-esRAGE group were significantly higher than control group (1542.13 ± 189.138 versus 1.01 ± 0.109, *P* < 0.001) (Figure 1(b)). Furthermore, the protein levels of esRAGE in supernatants (ELISA) in the esRAGE lentivirus transfection group were significantly higher than control group (*P* < 0.001) (Figure 1(c)). These findings indicated that lentiviruses overexpressing esRAGE were successfully transfected into HUVEC.

### 3.2. Effects of AGE-BSA and High esRAGE Expression on HUVEC Apoptosis

**Figure 2:**
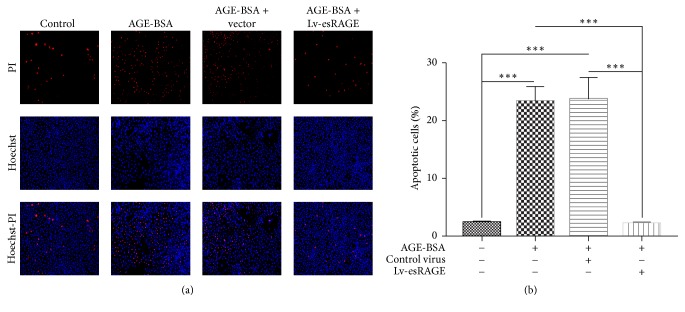
Effects of esRAGE on AGEs associated HUVEC apoptosis. Endothelial cells were cultured with complete medium containing 200 *μ*g/mL AGE-BSA for 24 h, submitted to the Hoechst-PI double-staining method, and photographed under a fluorescence microscope using the ZEN software. Total and apoptotic cells were counted using the Image-Pro-Plus 6.0 software, based on which apoptotic rates were calculated. (a) Cell staining in the 4 HUVEC groups, imaged by fluorescence microscope after Hoechst-PI staining. (b) Apoptotic rates in the 4 HUVEC groups. ^*∗∗∗*^*P* < 0.01.

AGE-BSA at 3 different concentrations (50, 100, and 200 *μ*g/ml) was added to endothelial cell cultures for 24 h. The apoptosis rate was concentration-dependently increased (data not shown). In subsequent experiments, 200 *μ*g/ml AGE-BSA was used to induce endothelial cell apoptosis. BSA had no effect on cell apoptosis. Therefore, the BSA culture group was not further assessed. Compared with the control group, AGE-BSA treated cells showed significantly increased apoptosis (*P* < 0.001) (Figures 2(a)-2(b)), and no significant difference between the AGE-BSA and AGE-BSA + control virus groups was obtained (*P* = 0.839). However, apoptosis was significantly decreased in the AGE-BSA + Lv-esRAGE group compared with AGE-BSA treated cells (*P* < 0.001) (Figures 2(a)-2(b)).

### 3.3. esRAGE Alleviates AGEs Induced Apoptosis

**Figure 3:**
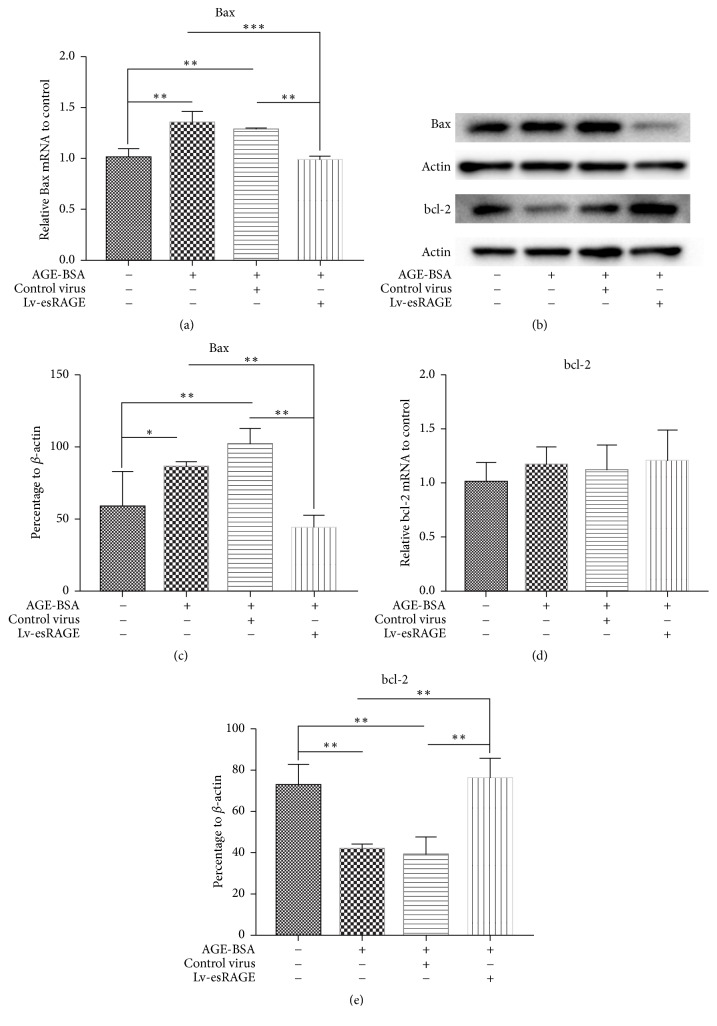
Effects of esRAGE on Bax and Bcl-2 levels in HUVEC. (a) Bax mRNA expression levels in the 4 HUVEC groups; (b) Western blot for Bax and Bcl-2 protein detection in the 4 HUVEC groups; (c) Bax protein expression levels in the 4 HUVEC groups; (d)-(e) Expression levels of Bcl-2 mRNA (d) and protein (e) in the 4 HUVEC groups.^*∗*^*P* < 0.05,^*∗∗*^*P* < 0.01, and ^*∗∗∗*^*P* < 0.001.

Compared with control group, Bax mRNA levels in the AGE-BSA group were significantly elevated (*P* = 0.001) (Figure 3(a)). There was no statistically significant difference between the AGE-BSA and AGE-BSA + control virus groups (*P* = 0.322). Bax mRNA levels were significantly reduced in the AGE-BSA + Lv-esRAGE group compared with AGE-BSA treated cells (*P* < 0.001) (Figure 3(a)). Compared with control group, BAX protein expression levels in the AGE-BSA group were markedly increased (*P* = 0.048) (Figures 3(b) and 3(c)). Meanwhile, there was no statistically significant difference between the AGE-BSA and AGE-BSA + control virus groups (*P* = 0.221). However, Bax protein levels were significantly lower in the AGE-BSA + Lv-esRAGE group compared with AGE-BSA treated cells (*P* = 0.007) (Figure 3(c)), suggesting that esRAGE inhibited AGEs induced Bax upregulation.

There were no statistically significant differences in Bcl-2 mRNA levels among the 4 groups (Figure 3(d)). Compared with control group, Bcl-2 protein levels in the AGE-BSA group were significantly reduced (*P* = 0.002) (Figures 3(b) and 3(e)). There was no statistically significant difference between the AGE-BSA and AGE-BSA + control virus groups (*P* = 0.704). However, Bcl-2 protein levels in the AGE-BSA + Lv-esRAGE group were significantly higher compared with those of AGE-BSA treated cells (*P* = 0.001) (Figure 3(e)), suggesting esRAGE inhibited AGEs associated Bcl-2 downregulation.

### 3.4. esRAGE Inhibits AGEs Induced NF-кB Upregulation

**Figure 4:**
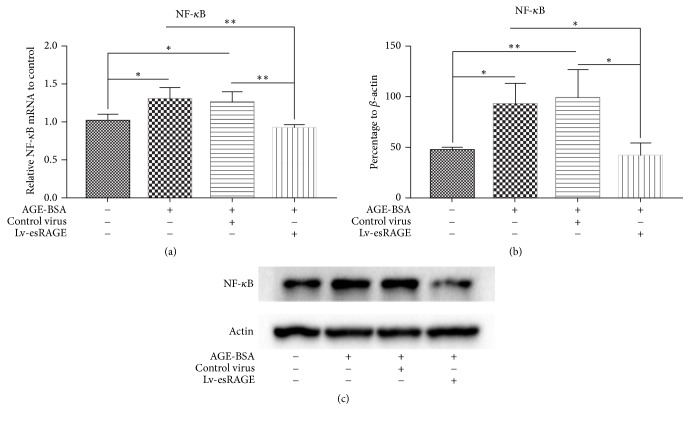
Effects of esRAGE on mRNA and protein expression levels of NF-кB in HUVEC. (a) Expression levels of NF-кB mRNA after treatment with AGE-BSA. (b) NF-кB protein expression levels after incubation with AGE-BSA. (c) NF-кB protein bands in the 4 HUVEC groups as determined by immunoblot. ^*∗*^*P* < 0.05; ^*∗∗*^*P* < 0.01.

Compared with control group, NF-кB mRNA levels in the AGE-BSA group were significantly higher (*P* = 0.016) (Figure 4(a)); NF-кB protein levels were significantly increased by 97% (*P* = 0.018) (Figure 4(b)). There were no statistically significant differences between the AGE-BSA and AGE-BSA + control virus groups, in the mRNA (*P* = 0.690) and protein (*P* = 0.709) expression levels. Compared with those of the AGE-BSA group, NF-кB mRNA levels in the AGE-BSA + Lv-esRAGE group were significantly lower (*P* = 0.004) (Figure 4(a)), as well as protein amounts (*P* = 0.01) (Figures 4(b)-4(c)). These findings suggested esRAGE inhibited AGEs induced NF-кB upregulation.

## 4. Discussion

This study demonstrated that, as a decoy receptor, esRAGE reduced AGEs induced apoptosis in HUVEC. Upregulating the antiapoptotic factor Bcl-2 and downregulating the proapoptotic factor Bax as well as the proinflammatory cytokine NF-*κ*B were involved in esRAGE protecting endothelial cells from AGEs induced cell apoptosis.

In this study, AGE-BSA was used to treat HUVEC, and NF-*κ*B and Bax expressions were significantly increased, which finally led to increased endothelial cell apoptosis. Interestingly, the antiapoptotic factor Bcl-2 showed reduced expression at the protein level while mRNA levels had no difference, suggesting AGEs/RAGE might be involved in posttranscriptional regulation of Bcl-2. In agreement, posttranscriptional regulation of Bcl-2 has been reported during apoptosis of endothelial cells [30]. However, the mechanism underlying AGEs associated apoptosis remains unclear and deserves further exploration. It has been reported that PI3K/AKT signaling and cGMP/NO activation participate in endothelial cell apoptosis [7, 31]. Meanwhile, posttranscriptional regulation by microRNAs, such as miR200b, miR200c, and miR214, also contributes to apoptosis [32, 33]. Incubation with AGEs may increase RAGE expression and expand the damaging effects of AGEs [8]. Taken together, previous studies and ours indicated that AGEs could induce apoptosis in endothelial cells.

In the present study, HUVEC overexpressing esRAGE significantly reduced apoptosis in endothelial cells by downregulating NF-*κ*B and Bax, while upregulating Bcl-2, conferring protective effects in endothelial cells. Recently, multiple clinical reports on esRAGE and vascular diseases have been published, but with controversial findings. Our previous clinical trial found that low plasma esRAGE levels are correlated with arteriosclerosis in type 2 diabetes [19]. In addition, Piarulli et al. used color-Doppler ultrasound to evaluate plaque lesions of the carotid artery, abdominal aorta, iliac artery, and lower limb blood vessels in type 2 diabetes; the results indicated that low esRAGE is associated with plaque formation in arteries [21]. Furthermore, Lu et al. suggested that low esRAGE is a risk factor for type 2 diabetes associated in-stent restenosis [34]. Moreover, Katakami et al. demonstrated that plasma esRAGE level is negatively correlated with carotid intima-media thickness (IMT) in patients with type 1 diabetes [20]. In patients with prediabetes or metabolic syndrome, esRAGE protects from arteriosclerosis [17, 22]. All the above clinical findings indicate that esRAGE could protect from diabetes associated chronic complications caused by vascular diseases, with endothelial cell injury representing an initiating factor in diabetic vascular disease. However, other clinical studies questioned the protective effects of esRAGE on blood vessels. For instance, Heier et al. conducted a 5-year follow-up observation of type 1 diabetic children and found that esRAGE and sRAGE do not protect from arteriosclerosis; intriguingly, individuals with high esRAGE and sRAGE levels showed higher incidence of arteriosclerosis in the diabetes group [24]. Similarly, Colhoun et al. analyzed 2838 type 2 diabetic patients and found no associations of esRAGE and sRAGE with stroke; however, high esRAGE and sRAGE levels were shown to predict cardiovascular disease (CHD) [25]. Furthermore, Yang et al. used 18F-fluorodeoxyglucose positron emission tomography to evaluate arteriosclerosis and reported that esRAGE in type 2 diabetics has no correlation with arteriosclerosis [23]. Therefore, the protective effects of esRAGE on blood vessels remain controversial based on discrepant clinical findings. In this study, we used HUVEC as a cell model and overexpressed esRAGE by the lentiviral method. At the cellular level, esRAGE reduced endothelial cell apoptosis and inflammation, confirming its protective effect on endothelial cells. These findings provide a theoretical basis for the future application of esRAGE in vascular diseases. Certainly, more in vivo trails need to be conducted to confirm the protective role of esRAGE on the arteriosclerosis, especially in diabetic animal models and patients. Furthermore, more exploration about the mechanisms and cellular signal pathways of esRAGE should be performed in future.

## 5. Conclusions

In conclusion, the present study demonstrated that esRAGE protects endothelial cells from AGEs associated apoptosis, via Bax and NF-кB downregulation and Bcl-2 upregulation. Further studies are required to confirm these findings.
